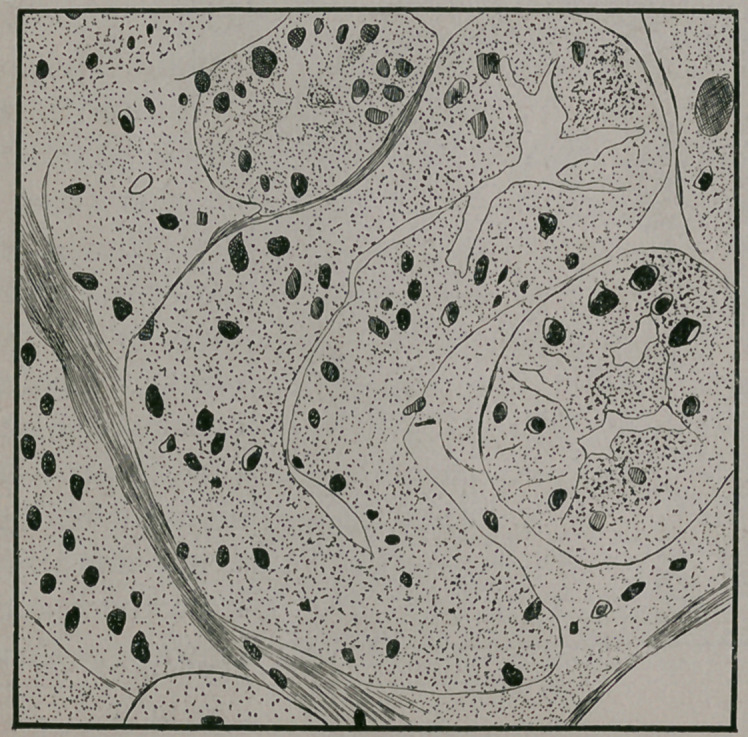# Acute Parenchymatous Nephritis

**Published:** 1885-10

**Authors:** A. W. Clement

**Affiliations:** Montreal Veterinary College


### Art. XXIII.—ACUTE PARENCHYMATOUS NEPHRITIS.

HÆMOGLOBINURIA–TOXICA. BOLLINGER. AZOTUREA. WILLIAMS.

BY A. W. CLEMENT, V.S.,
Montreal Veterinary College.

Probably there is no disease met with in veterinary practice concerning which there are so many conflicting opinions as to the lesions and the genesis of those lesions, as in that affection usually described under the heading of Hæmoglobinuria.

A few consider the primary lesions to be located in the kidneys, while the majority, though admitting structural changes in those organs, look for the primary changes in the blood itself, and maintain that only through the irritating influence of this poison do the kidneys become involved.

Certainly the fact that changes of a like character to those found in the kidney are found in the liver and certain muscles, together with the very violent symptoms, would lead us to suppose that an intense irritant existed in the blood, but might not this be a consequence of structural changes in the kidney? Would not an attack of acute parenchymatous nephritis with uraemic poisoning bring on similar conditions which would be manifested by much the same symptoms? I think it would.

I can not agree with those who affirm that red blood corpuscles are very rarely to be found in the urine in this disease, and I make this statement not altogether on my own authority, for my attention was first called to their presence in the urine by Dr. Osler some three years since. I remember the case in point very well. The urine was dark and quite ammoniacal. A few well defined corpuscles could be seen, while many more, which were decolorized and more or less degenerated, could be made out by close observation, as well as hyaline casts and degenerated renal epithelium. The post-mortem examination proved the correctness of this observation, for, while the kidneys were dark and swollen, points of hæmorrhage could be seen here and there in the cortex, and examination of hardened sections with a No. 7 Hartnack showed ruptured capillaries with extravasation into the interstitial tissue, in some places completely plugging the tubules. The red corpuscles were very well defined. Since then I have seen these degenerated corpuscles several times in the urine, though not in every case, and have seldom failed to find the distended capillaries and extravasation of blood in the tissues.

It is not at all strange that the corpuscles should appear so much better defined in the kidney than in the urine if we accept the statement of most writers on such subjects, for nearly all agree with Roberts, who, in his work on Urinary and Renal diseases article on Hæmaturia, says that "The corpuscles quickly disappear if the urine be of a low specific gravity or ammoniacal." That the urine is ammoniacal in this disease is very well known, and when we consider that while the flow into the bladder is continuous, the outflow is periodical, and in most of these cases dependent upon surgical interference, is it not possible that we are dealing with a Hæmaturia, and that only after its entrance into the bladder does it become a hæmoglobin urea.

If Franck, whom Mr. Billings quotes in his article on Hæmoglobinuria-toxica, has demonstrated beyond a doubt that the urine has no solvent action on hæmoglobin in the corpuscle, obviously no such change could have occurred in the bladder; but his statement does not agree with any which I have seen, and is not, in my opinion, borne out by experimental evidence.

If, on the other hand, our observations were correct, if degenerated corpuscles were seen in the urine in a given case, and if, on the microscopical examination of sections of the kidney in the same case, we found congestion, rupture, extravasation, and plugging of the tubules with blood casts, and that in the organ the corpuscles were much better defined and of a more natural color, it is evident that we have a condition resembling hæmaturea rather than hæmoglobinuria.

If, in addition to this we found the parenchyma degenerated, and in some cases an increased amount of connective tissue, we may conclude that we have to deal with acute hæmorrhagic nephritis.

Case No. I.—A gelding, of about 1,400 lbs. weight, used to hard work, but very well cared for and highly fed, remained idle for two days. On the third day he was put to his regular work, but when about half a mile from the stable the usual symptoms came on. After staggering for about a rod he fell and was unable to rise. He was removed to the stable on a truck. It was nearly an hour before surgical assistance arrived. Then about two quarts of very dark colored urine, having a very ammoniacal odor, was taken from him. Tested with Nitric Acid, a dense precipitate was thrown down, which, examined microscopically, was found to be composed in large part of nitrate of urea. Specimens of the urine as drawn from the bladder were found to contain degenerated renal epithelium but no blood cells, though many coarse granules and degenerated bladder epithelial cells were found

AUTOPSY—Made four hours after death.—All the organs dark in color. Kidneys swollen and soft, capsule easily removed. On section the substance of the organ had a clouded appearance, with here and there in the cortex small points of hæmorrhage. Bladder nearly empty, and the mucous membrane very dark in color. Considerable infiltration of the connective tissue, especially about the kidneys. Liver somewhat enlarged and fatty. Spleen engorged. Intestine comparatively pale. Lungs dark in color, but crepitant throughout. No accumulation of mucous in the bronchial tubes. Structure of the heart normal. Left chambers empty; right chambers full of blood which was only partially coagulated. Usual appearance of the muscles of the haunch and thigh. The striæ completely obliterated in some of the fibres, while others were comparatively normal. A finely granular material took the place of the striæ where the sarus elements were wanting.

Sections of kidney, which had been hardened in Mueller's fluid, and stained with hæmotoxyline, when examined, showed great engorgement of the capillaries and extravasation of red blood corpuscles into the interstitial tissue, which was somewhat increased, and in which many nuclei could be plainly seen In some places the cells being so much swollen as to entirely fill up the lumen, while in others the lumen was plugged with finely granular casts, in some of which red blood corpuscles could be seen indicated the existence of acute parenchymatous degeneration.

FIG. 1.—*a*. Showing tubule with blood casts and a few free epithelial cells.

Diseased portions of the liver showed the cells to be extremely granular, so much so as to obliterate the nuclei in some places. The duration of the malady was six hours.

CASE NO. II.—Nothing special about the history. The duration of the malady was three days. Urine very dark from the first. Microscopic examination showed hyaline and blood casts, with some degenerated free red blood corpuscles. The interstitial tissue was more hypertrophied than in Case No. 1, and the nuclei were more numerous, so that in some places it pressed upon the tubules, lessening the calibre of their lumena.

Parenchyma swollen and very granular, completely obliterating the nuclei of many of the cells. In some of the tubules the cells were so swollen as to completely fill the tubule with one mass of granulous protoplasm in which no traces of nuclei could be seen, while in others the cells were better defined and the lumen plugged with blood casts. There was great distention of the capillaries, with rupture of arterioles in one or two places, the nuclei in the muscular layer of the wall showing very distinctly.

CASE NO. III.—The subject in this case was a Bay Gelding of about 1,300 lbs. weight, used to working in an express wagon· The owner intended to sell him and for the past few weeks had given him an extra quantity of food. He remained in the stable for two days, and on the third day was attacked soon after leaving the stable. He was catheterized about two hours later and a quantity of very dark colored urine came away. When examined some three hours later nothing but granule masses could be seen, no blood corpuscles could be detected, nor could any cylinders be seen.

AUTOPSY—Four hours after death.—On the skin being removed the usual streaked appearance of the gluteals could be well seen. All the internal organs were dark in color.

Kidneys soft and swollen. On removing the capsule small pieces of the substance came away with it. On section the usual cloudy appearance was met with.

As in cases I and II points of hæmorrhage could be seen in the cortex, but the rest of the cortical portion seemed more anæmic than in the other cases. The tubules stood out very prominently.

The liver softer than normal and fatty. There was great œdema of the connective tissues of the body. The meninges in the lumbar region of the cord greatly congested.

Microscopic examination of the kidneys showed no capillary congestion. No blood vessels to be seen in this section. The cells of the tubules were so degenerated as to be unrecognizable as such, and in their place was one continuous mass of granular protoplasm with which some of the tubules were completely filled while in others this granular mass was a little less condensed in the center, so that the outline of the lumen could be made out.

As will be seen by the accompanying drawing there was considerable difference in the density of the granular masses in the different tubules, some were quite dark, while others were much lighter.

The fibres of the interstitial tissue were on the stretch, leaving considerable spaces between them, which were more or less studed with granules.

CASE NO. IV.—Dark Brown Gelding 1,600 lbs. weight. The point of interest in the clinical history of this case is, that though the paralytic symptoms were very marked from the onset, yet, though two hours had elapsed, when the urine was drawn from the bladder it was perfectly clear and nearly neutral in reaction ; and when the catheter, was again passed some three hours later, a small amount of but slightly tinged urine was obtained, in which microscopically a few red corpuscles could be seen. Nitric acid threw down a slight blood floculent precipitate which did not disappear on boiling. At the third catheterization, however, the urine was very dark, had a strong ammoniacal odor and contained some much degenerated corpuscles and granular masses but no cylinders. In this case the urine remained normal in color for at least two hours, yet the paralysis was so complete that the animal from the very commencement of the attack had no power whatever in the hind quarters. He would strike out wildly with the fore limbs, but the hind legs remained perfectly motionless.

AUTOPSY—Made two hours after death.—Kidneys swollen and soft. On section, points of hæmorrhage were to be seen here and there, while the remaining parenchyma was quite anæmic. The tubules were very distinct. Bladder empty. Liver quite firm, but a little fatty. Excessive œdema of the connective tissue and of the muscles, especially the gluteals. Congestion of the spinal meninges, especially in the posterior dorsal and lumbar regions. On microscopic examination of the kidneys in hardened sections, the only lesions seen were in the greatly swollen and extremely granulous condition of the tubal epithelium, completely filling many of the tubes with one mass of granular protoplasm with nuclei scattered here and there,and a somewhat hypertrophied condition of the interstitial tissue in places, while in others the tubes were so distended as to press upon each other with no recognizable intervening tissue. The anæmic muscle fibers showed the usual granular deposit in place of the striæ.

### CONCLUSIONS.

I.—That the lesions in the kidneys are identical with those of acute nephritis in man.

II.—That the secreting power of the cells had become gradually diminished from over stimulation due to a hyper-nitrogenous condition of the blood.

III.—That some excitant, such as cold or exercise after a period of quiet, gave rise to an increased flow of blood to the already overworked organ, thereby causing a complete suppression of secretion, with consequent exudation and hæmorrhage followed by uræmic poisoning.

IV.—That inasmuch as degenerated red blood corpuscles are found in the urine, and quite well defined ones are to be found in the blood vessels and tubules, it is not hæmoglobin-uria, which is secreted by the kidneys but rather is it hæmaturea, which under the influence of the alkaline urine in the bladder is there converted into hæmoglobin-uria.

V.—That with our present lack of information concerning the microscopic appearances of the cord, in these cases, we would not be justified in offering an opinion as to the cause of the paralysis.

---

NOTE.—The drawings were kindly made by MR. J. H. Y. GRANT, medical student, McGill College.

## Figures and Tables

**Fig. 1. f1:**
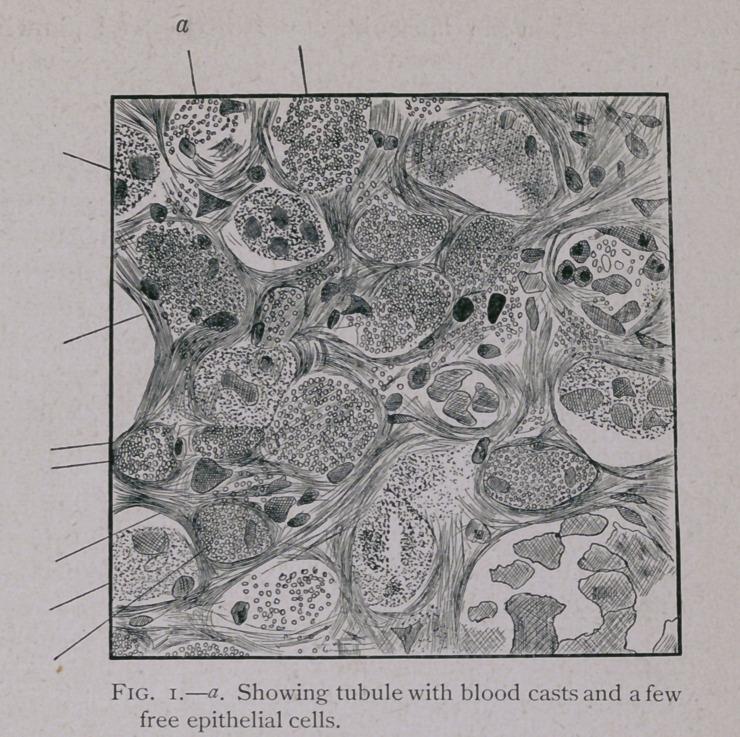


**Fig. 2. f2:**
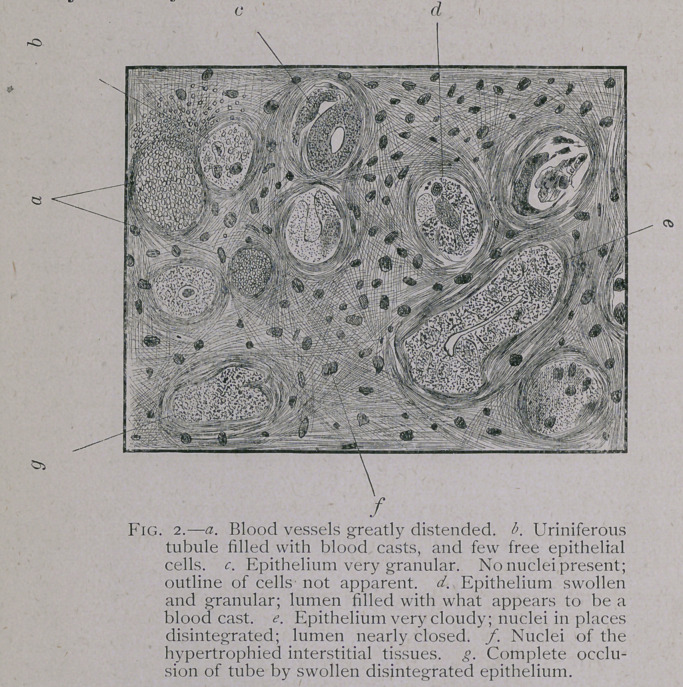


**Figure f3:**
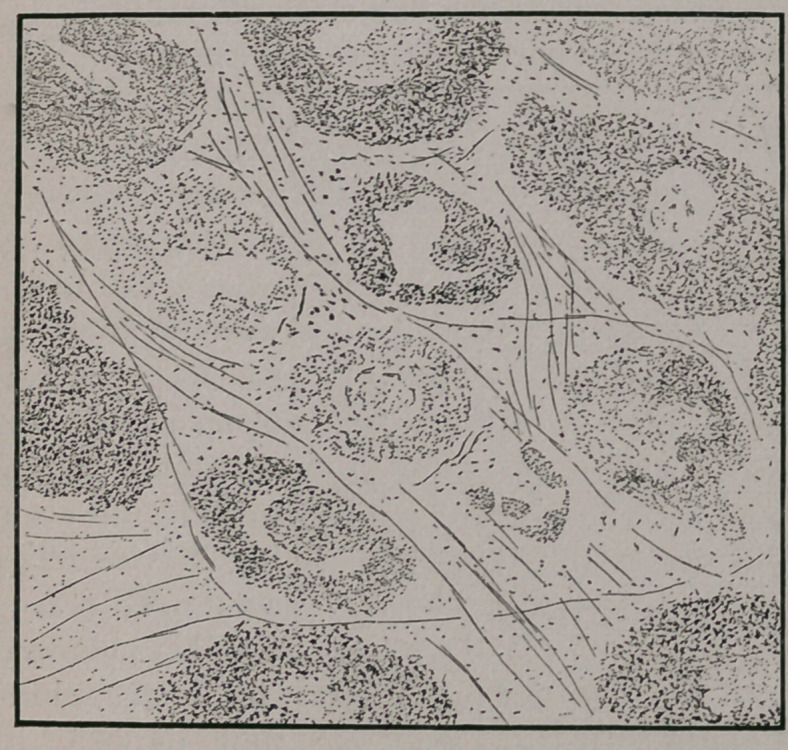


**Figure f4:**